# A rare case of submucosal foreign body in the duodenal bulb

**DOI:** 10.1055/a-2686-7898

**Published:** 2025-09-11

**Authors:** Weizhao Wang, Mengxian Ju, Bin Deng, Jun Liu, Weiwei Han, Xiaoyin Wang, Chao Sun

**Affiliations:** 1 370089 Department of Endoscopy Center, Clinical Medical College of Yangzhou University, Northern Jiangsu People’s Hospital, Yangzhou, China; 2 370089 Department of Endocrinology, Clinical Medical College of Yangzhou University, Northern Jiangsu People’s Hospital, Yangzhou, China; 3 370089 Department of Anesthesiology, Clinical Medical College of Yangzhou University, Northern Jiangsu People’s Hospital, Yangzhou, China

A 59-year-old woman was hospitalized with intermittent upper abdominal pain of 2 months’ duration. She was previously in good health. Physical examination revealed epigastric tenderness without rebound pain. Her complete blood test, liver, renal, coagulation function, and tumor markers were within reference values.

Gastroscopy at an outside hospital 1 month ago suggested chronic gastritis with no abnormalities in the duodenal bulb. After admission, abdominal CT showed a 13.9-mm-long spindle-shaped high density in the duodenal bulb. A foreign body (FB) was considered ( Fig. 1 ). Gastroscopy showed a 15 mm × 15 mm mass in the duodenal bulb, mucosal edema, and a small patchy ulcer on the surface ( Fig. 2 ). Ultrasound gastroscopy showed a hyperechoic space with a cross-section of approximately 2 mm × 2 mm in the submucosal layer and muscularis propria of the duodenal bulb mass ( Fig. 3 ). The patient was diagnosed with a submucosal FB in the duodenal bulb, After obtaining the patient’s consent, we decided to perform a transgastroscopic mucosal incision to remove the FB ( Video 1 ). After performing the mucosal incision, we visualized a bar-shaped bony FB buried in the mass ( Fig. 4 ). After sufficient separation of the FB, the FB was removed using a FB forceps, which was approximately 14 mm in length ( Fig. 5 ), and the wound was finally closed with metal clips.

**Fig. 1:**
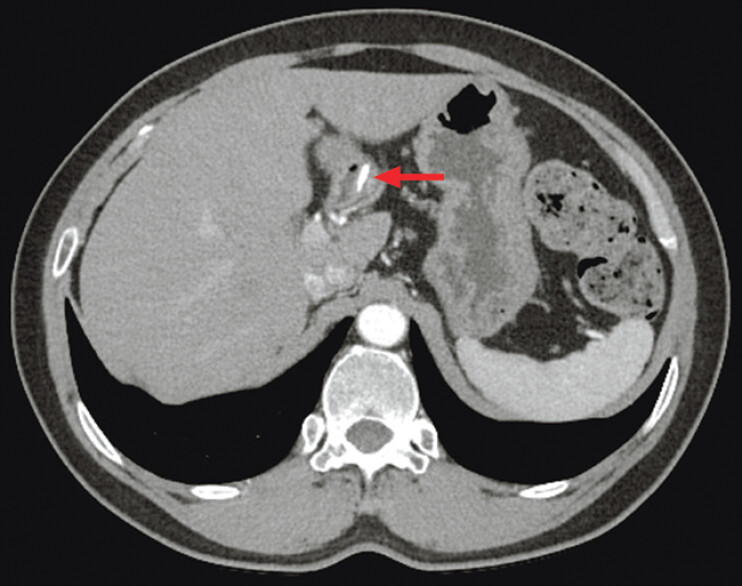
Abdominal CT showed a 13.9-mm-long spindle-shaped high density in the duodenal bulb.

**Fig. 2:**
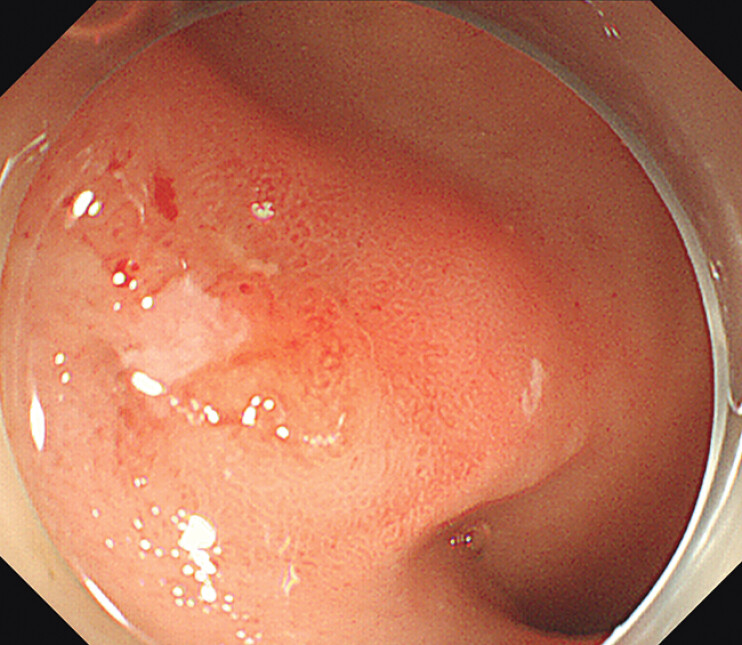
Gastroscopy showed a 15 mm × 15 mm mass in the duodenal bulb, mucosal edema, and a small patchy ulcer on the surface.

**Fig. 3:**
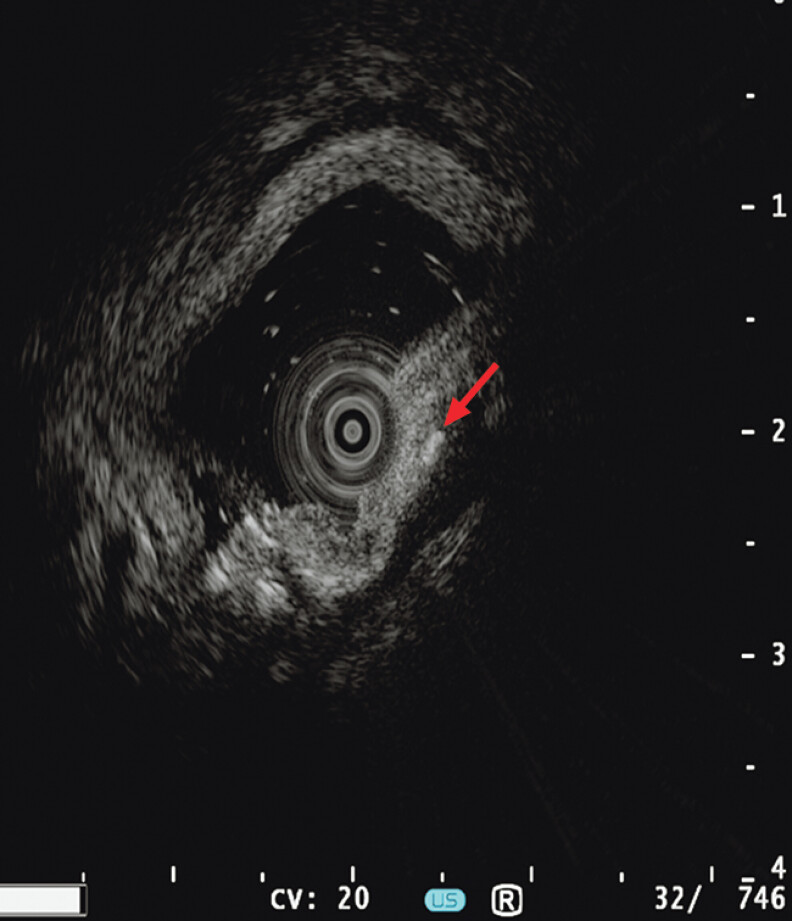
Ultrasound gastroscopy showed a hyperechoic space with a cross-section of approximately 2 mm × 2 mm in the submucosal layer and muscularis propria of the duodenal bulb mass.

After the gastroscope reached the location of the duodenal bulb mass, a mucosal incision was performed and a bar-shaped bony FB was found buried within the mass. After adequate separation of the FB, the FB was removed, which was approximately 14 mm in length, and the wound was finally closed with metal clips.Video 1

**Fig. 4:**
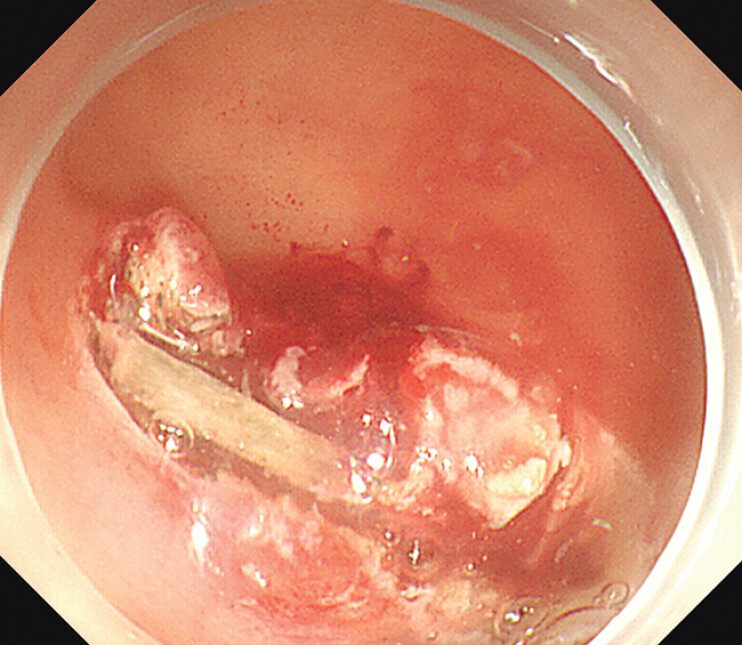
After performing the mucosal incision, we visualized a bar-shaped bony FB buried in the mass.

**Fig. 5:**
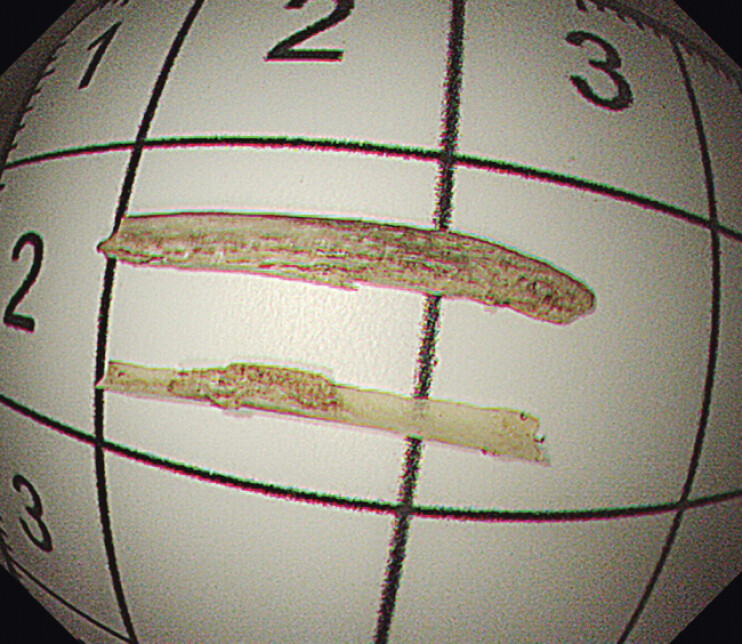
Bone FB was successfully removed, length about 14 mm.

We removed the bone FB intact with no complications. She was then questioned and had no memory of swallowing the bone and was successfully discharged home after conservative treatment. No symptoms were found during the three-month follow-up, and she was unwilling to repeat the gastroscopy and CT.

Bone FB in the duodenal bulb is common 1 2 , but submucosal FB in the duodenal bulb is very rare, and we have searched a large number of literature to find almost no similar cases. Bone FB in this area often leads to bleeding, perforation, and abscess formation and can usually be removed endoscopically 3 , but in combination with perforation, peritonitis, severe bleeding, and abscess, surgical removal of the FB is often required 1 . Our case is a submucosal FB in the duodenal bulb, with a residence time of more than 2 months, only abdominal pain manifestation, insidious symptoms, and endoscopic observation cannot be detected, and the diagnosis is difficult and needs to be jointly diagnosed by CT, ultrasound endoscopy, etc. For treatment, the removal of the FB after incision of the mucosal layer by gastroscopy is still preferred by this kind of patients, which is safe and effective, and can be used as a reference for clinicians.

Endoscopy_UCTN_Code_TTT_1AO_2AL

## Notes

Endoscopy E-Videos https://eref.thieme.de/e-videos
*E-Videos* is an open access online section of the journal *Endoscopy* , reporting on interesting cases and new techniques in gastroenterological endoscopy. All papers include a high-quality video and are published with a Creative Commons CC-BY license. Endoscopy E-Videos qualify for HINARI discounts and waivers and eligibility is automatically checked during the submission process. We grant 100% waivers to articles whose corresponding authors are based in Group A countries and 50% waivers to those who are based in Group B countries as classified by Research4Life (see: https://www.research4life.org/access/eligibility/ ). This section has its own submission website at https://mc.manuscriptcentral.com/e-videos .
